# Classifying Sustainable Development Goal trajectories: A country-level methodology for identifying which issues and people are getting left behind

**DOI:** 10.1016/j.worlddev.2019.06.031

**Published:** 2019-11

**Authors:** John W. McArthur, Krista Rasmussen

**Affiliations:** a Brookings Institution, United States; b United Nations Foundation, United States

**Keywords:** Sustainable Development Goals, United Nations, Methodology, Poverty, Mortality, Canada

## Abstract

How useful are the Sustainable Development Goals for conducting empirical analysis at the country level? We develop a methodological framework for answering this question, with special emphasis on the SDGs’ normative ambition of “no one left behind.” We first classify all 169 SDG targets and find that 78 incorporate an outcome-focus that is quantitatively assessable at the country level, including 43 through a systematic approach to establishing “proxy targets.” We then present a framework for diagnosing the embedded diversity of absolute and relative indicator trajectories in a harmonized manner, based on a country’s share of its starting gap on course to be closed by the relevant deadline. In turn, we present a method for estimating the human consequences of falling short on targets, measured by the number of lives at stake and people’s basic needs at stake.

As a case study, we apply the framework to Canada, an economy not commonly examined in the context of global goals. We are able to assess a total of 61 targets through the use of 70 indicators, including 28 indicators drawn from the United Nations’ official database. Overall, we find Canada is on course to succeed on 18 indicators; to cover at least half but less than the full objective on 7 indicators; to cover less than half the required distance on 33 indicators; and to remain stagnant or move backwards on 12 indicators. Among indicators assessed, the country is only fully on track to achieve one SDG. Shortfalls suggest approximately 54,000 Canadian lives at stake and millions of people left behind on issues like poverty, education, intimate partner violence, and access to water and sanitation. Our diagnostic framework enables considerable, if only partial, quantification of a country’s SDG challenges, recognizing the wide range of contexts for underlying data availability and societal problems.

## Highlights

• A harmonized methodology can translate SDG targets into an empirical framework pertinent to each country’s challenges.
• Of 169 SDG targets, 35 include quantified and measurable country-level outcomes; 43 are assessable through proxy targets.
• A clear SDG diagnostic framework can distinguish between policies that are succeeding and those meriting a new approach.
• A methodological focus on “no one left behind” suggests even Canada is only fully on track for one of the 16 SDGs assessed.
• The human scale of SDG shortfalls can be estimated; Canada has approximately 54,000 lives at stake.

## 1 Introduction

Since their adoption at the United Nations (U.N.) in 2015, the Sustainable Development Goals (SDGs) have gained increasingly widespread traction as a normative policy framework. But what is the empirical relevance of the goals? A headline in the elite Economist newspaper once described the goals as “worse than useless,” criticizing the substantive breadth and rhetoric embedded across 169 targets spanning 17 diverse policy realms (“The 169 Commandments,” 2015). At the country level, it is not *ex ante* clear how useful the global political framework is for conducting empirical analysis relevant to the lives of real people. Our paper considers this question, with a special emphasis on the SDGs’ stated ambition of “no one left behind.”

There are many analytical challenges embedded in translating the SDGs from diplomatic text to quantitative assessment. The goals and targets touch on a wide array of topics and disciplines, each of which is anchored in its own norms of measurement and reporting, making it difficult to distill trends and gaps in a standardized manner across issues. Moreover, there is no overarching empirical logic guiding all the goals. Target ambitions range from the absolute universal elimination of one problem to a proportionate domestic reduction of another. Meanwhile, many targets are quantitatively ambiguous or focused on process ambitions rather than policy outcomes. Uncertainty regarding a target’s intrinsic empirical aspirations risks hindering that target’s efficacy in helping to stimulate improvements in policy action.

Our methodology addresses these challenges by producing a multi-step analytical framework to translate the SDGs from a U.N. framework to a country-level diagnostic tool. Specifically, we consider three questions from the country-level perspective where sovereign policy decisions are made. First, which of the SDG targets lend themselves to quantitative assessment? Second, how can the information embodied in a vast range of SDG indicators be coherently synthesized to identify which issues are lagging? Third, how can such a diagnosis be interpreted in terms of absolute human consequences, measured by the number of people who will be left behind on each issue if the relevant target is not achieved? We then apply this framework to a case study of Canada, an economy not commonly examined in the context of global goals.

Our methodology uses a multi-step logic that aims to be applicable to any country and, subject to data availability, at the subnational level too. We attempt a “by-the-book” approach that follows the U.N.’s formal SDG architecture of goals, targets, and indicators as much as practical. In cases where the official U.N. language is quantitatively vague, we develop a logic of “proxy targets” to enable empirical assessment where viable. One value of this framework is that it allows a straightforward and transparent methodology for translating the normative aspirations of the SDGs into specific estimates of the consequences of falling short on the targets. It also helps draw attention to population-specific data gaps and policy gaps. Whereas some studies have interpreted the SDGs as a scale for comparing progress across countries (OECD, 2017, Sachs et al., 2018), our approach considers the extent to which the SDGs can be implemented as a tool for tracking progress within countries, relative to each society’s own needs on each issue.

The remainder of the paper is presented in four sections. Following this introduction, section two describes previous studies relevant for domestic SDG assessment. Section three presents the framework’s key methodological elements. Section four presents the framework results when applied to Canada. Section five concludes.

## 2 Literature review

A number of studies have conducted initial attempts at translating the intergovernmental SDG targets into empirical assessments. Much of the early SDG benchmarking research takes stock of starting baselines across countries and issues (United Nations, 2018, World Health Organization, 2017, World Bank, 2018). Other studies have considered questions of SDG target sorting and empirical benchmarking. For example, the Organization for Economic Cooperation and Development (OECD, 2017) identifies targets in which the outcome level is specified in the target language and then categorizes targets based on whether this outcome is defined in the same absolute terms for all countries or defined relative to each country’s starting baseline. It uses this target classification to assess domestic challenges across advanced economies. For SDG targets that are not quantified as written, the authors either substitute targets from other international agreements or set the relevant standard as the 90th percentile among OECD countries as of 2010.

Other studies have identified ways to systematize measures of progress across the goals. For example, Nicolai, Hoy, Berliner, and Aedy (2015) looks at aggregate global trajectories out to 2030 for a sample of 17 indicators, one for each goal. It classifies each indicator based on the share of distance the world travels toward the target if recent trends continue. The European Union (EU, 2017) uses short- and long-term trends to assess the EU’s aggregate performance on a selection of indicators. For indicators with quantified outcomes defined either in SDG target language or EU strategy, indicators are classified into four categories, using the ratio of recent rate of progress to rate required to reach the target. For indicators without quantified outcomes, indicators are categorized by recent rates of progress.

The Sustainable Development Solutions Network and Bertelsmann Stiftung (SDSN, Sachs et al., 2018) presents an important country-level trend analysis for a subset of indicators across goals and classifies each based on the share of distance traveled toward absolute global thresholds. To set values for target achievement, SDSN identifies a range of values bounded by the “technical optimum” (e.g. 100 percent access to basic sanitation) and an absolute threshold at which a country is deemed to have achieved the SDG, which is set at a different value (e.g. 95 percent access to basic sanitation). In situations where a country has surpassed the absolute threshold prior to the start of the SDG period, SDSN classifies them as having already met the target, including when the target language defines an outcome in relative terms. As an example of the latter, target 3.4 calls for a one-third reduction in pre-mature mortality from non-communicable diseases (NCD). SDSN classifies a country as having achieved the relevant standard if it is at or below an absolute threshold of 15 percent of 30-year-olds dying from cardiovascular diseases, cancer, diabetes or chronic respiratory diseases before their 70th birthday.

Some studies focus on specific sectoral issues. On extreme income poverty, for example, Cuaresma et al. (2018) presents country-level trajectories out to 2030. The Global Burden of Disease, 2017 Collaborators (2018) estimates country-level progress towards 2030 on specific health-related indicators. UNICEF (2018) looks at projections for a sample of child-focused indicators. McArthur, Rasmussen, and Yamey (2018) examines maternal mortality and child mortality trends and identifies the approximate number of “lives at stake” if each country continues on its recent trajectory.

Some national governments have included gap and trend analysis in their Voluntary National Reviews (VNR) presented at the U.N.’s annual SDG-focused High-Level Political Forum. Kindornay (2019) finds that 32 of 46 countries that presented in 2018 included some form of baseline or gap analysis. Some countries consider indicator trajectories. For example, Egypt’s VNR classifies multiple indicator trends per goal under three categories: positive change, negative change, and no change (Ministry of Planning, Monitoring, and Administrative Reform, 2018). Latvia similarly classifies recent trajectories but does so based on progress toward targets drawn from the SDGs and its own national sustainable development strategy (Cross Sectoral Coordination Centre of Latvia, 2018).

The current study builds on previous research in multiple ways. Our approach presents a standardized filtering logic for assessing several dozen targets across all countries and at subnational levels. Adhering to the formal SDG target language and framing as closely as possible, we offer a method for quantifying and classifying SDG target trajectories relative to each country’s own situation, where appropriate, rather than to global aggregates. Consistent with the SDG aim of “no one left behind,” this also includes a literal interpretation of universal coverage targets where relevant.

## 3 Methodology

Our analytical framework is comprised of five key steps. First, we identify which SDG targets are quantitatively assessable at the country level. Second, in cases where the official U.N. language is quantitatively vague, we present an approach for establishing “proxy targets.” Third, we present a decision tree logic for identifying relevant data sources. Fourth, we classify forward-looking trajectories of several dozen corresponding indicators into a harmonized analytical framework. Finally, for targets focused on human outcomes, we demonstrate how shortfalls in trajectories to 2030, or corresponding SDG target year, can be translated into approximate numbers of lives and people’s needs at stake.^1^ These steps are described further below and the Appendix includes target-specific details for readers interested in more detailed examination or replication of the results.

### 3.1 Identifying assessable, country-level SDG outcome targets

**Fig. 1:**
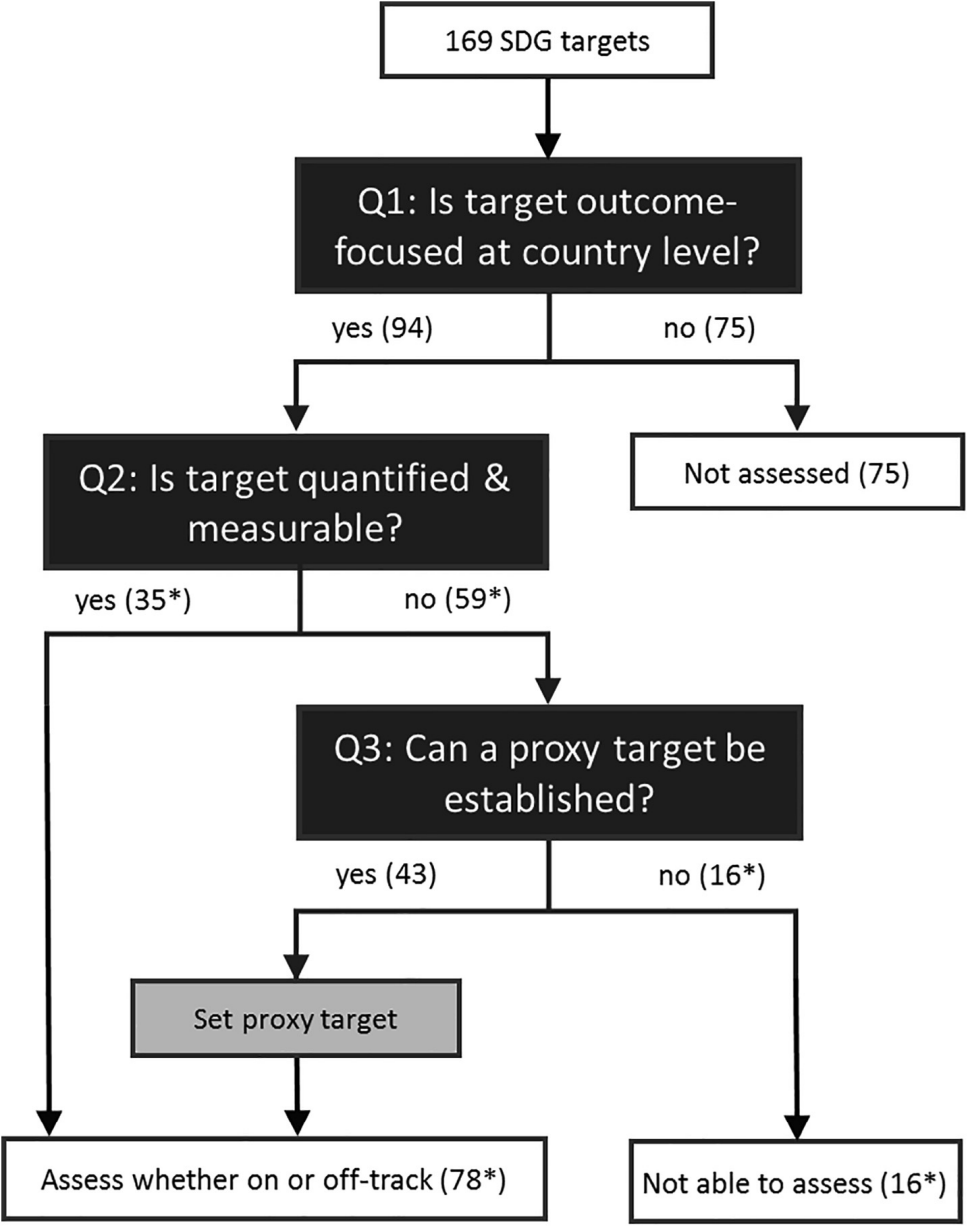
Logic for identifying assessable, country-level SDG targets. Note: * Numbers differ for the special case of least developed countries (LDCs). Targets 8.1 and 9.2 are quantified and measurable at the country level for LDCs. For all other countries, a proxy target cannot be established for these two targets. For LDCs, there are instead 37 quantified and measurable targets and 57 that do not pass the same test. Of those 57, the same 43 proxy targets can be established as for non-LDCs, yielding a total of 80 targets for which LDCs could potentially be assessed for on or off-track status and 14 targets for which they cannot. Source: Authors’ calculations.

To identify which targets can be used to assess progress, we use a filtering logic as outlined in Fig. 1. Of the 169 total SDG targets, we first identify those that are outcome-focused at the country level. An initial cut at this is provided by the official SDG framework, which distinguishes between outcome targets (numbered 1.1, 1.2, and so forth) and “means of implementation” (MOI) targets (lettered 1.a, 1.b, and so forth). Consistent with that structure, we filter out all “lettered” targets that focus on MOI. We also filter out all targets under Goal 17, a process-focused goal that seeks to “strengthen the means of implementation and revitalize the Global Partnership for Sustainable Development.”

We then examine the remaining numbered targets to confirm whether they are outcome-focused at the country level. We find that 13 numbered targets are not outcome-focused or assessable at the country-level and filter them out of the core sample, reducing the number of examined targets down to 94. Among those filtered out, target 15.9, for example, aims to, “integrate ecosystem and biodiversity values into national and local planning.” This focuses on policy planning, so we classify it as a MOI target rather than an outcome target. Similarly, target 10.5 aims to, “improve the regulation and monitoring of global financial markets and institutions,” which we deem not to be outcome-focused at the country level. Some category determinations inevitably entail a degree of subjectivity, so the Appendix reports relevant details on our classifications for all numbered targets.

The second branch of the decision tree of Fig. 1 shows how we next divide the 94 targets into two groups: those that are both quantified and measurable versus those that are not. Targets are considered quantified if their language includes either an explicit numerical target or an absolute verbal target, such as “conserve at least 10 percent of coastal and marine areas” (target 14.5) or “end hunger” (2.1), respectively. Targets are considered measurable if they have a clearly identifiable outcome and an objective direction for progress. For example, target 11.2 aims to “provide access to safe, affordable, accessible and sustainable transport systems for all.” We deem this to be conceptually quantified (i.e., access for all) but not measurable, because the target language is unclear as to how accessible and sustainable transport systems would be measured. Conversely, target 16.1 to “significantly reduce all forms of violence and related death rates everywhere” is conceptually measurable (i.e., the rate of violence should decline) but not quantified, since the amount of reduction to be achieved is unclear.

These distinctions guide the identification of 35 targets that are outcome-focused, quantified, and conceptually measurable for any country as written. We deem 27 of these to be absolute targets, applying the same outcome standard to all countries, such as ending extreme poverty or reducing child mortality to no more than 25 deaths per 1000 live births. We classify the other eight as relative targets, such as cutting domestic poverty by half or reducing non-communicable disease mortality by one-third, whereby each country’s outcome objective is set in relation to its 2015 starting point.^2^

Separately, we identify 59 targets where the official U.N. language is either not quantified or not measurable. For the special case of the least developed countries (LDCs), the corresponding breakdown at the second branch of the Fig. 1 decision tree adjusts to 37 and 57 targets, respectively, (rather than 35 and 59) since two targets are specifically quantified and measurable for the LDC context.^3^

### 3.2 Set proxy targets where relevant

The third branch in the Fig. 1 decision tree applies to the 59 targets for which the U.N. framework language is not adequately quantified and measurable to be numerically assessable. For these cases, we adopt an expansive “proxy target” approach, aiming to apply a consistent logic that can allow country-level progress to be assessed wherever data permit.

A first step in this direction is to identify any existing, equivalent national targets within the country of interest that are quantified and measurable. For example, target 7.2 is to “increase substantially the share of renewable energy” by 2030. The phrase “increase substantially” is quantitatively ambiguous, but in the Canadian context there is a relevant target in the country’s Federal Sustainable Development Strategy, “By 2030, 90% and in the long term, 100% of Canada’s electricity is generated from renewable and non-emitting sources” (ECCC, 2016).^4^

**Fig. 2:**
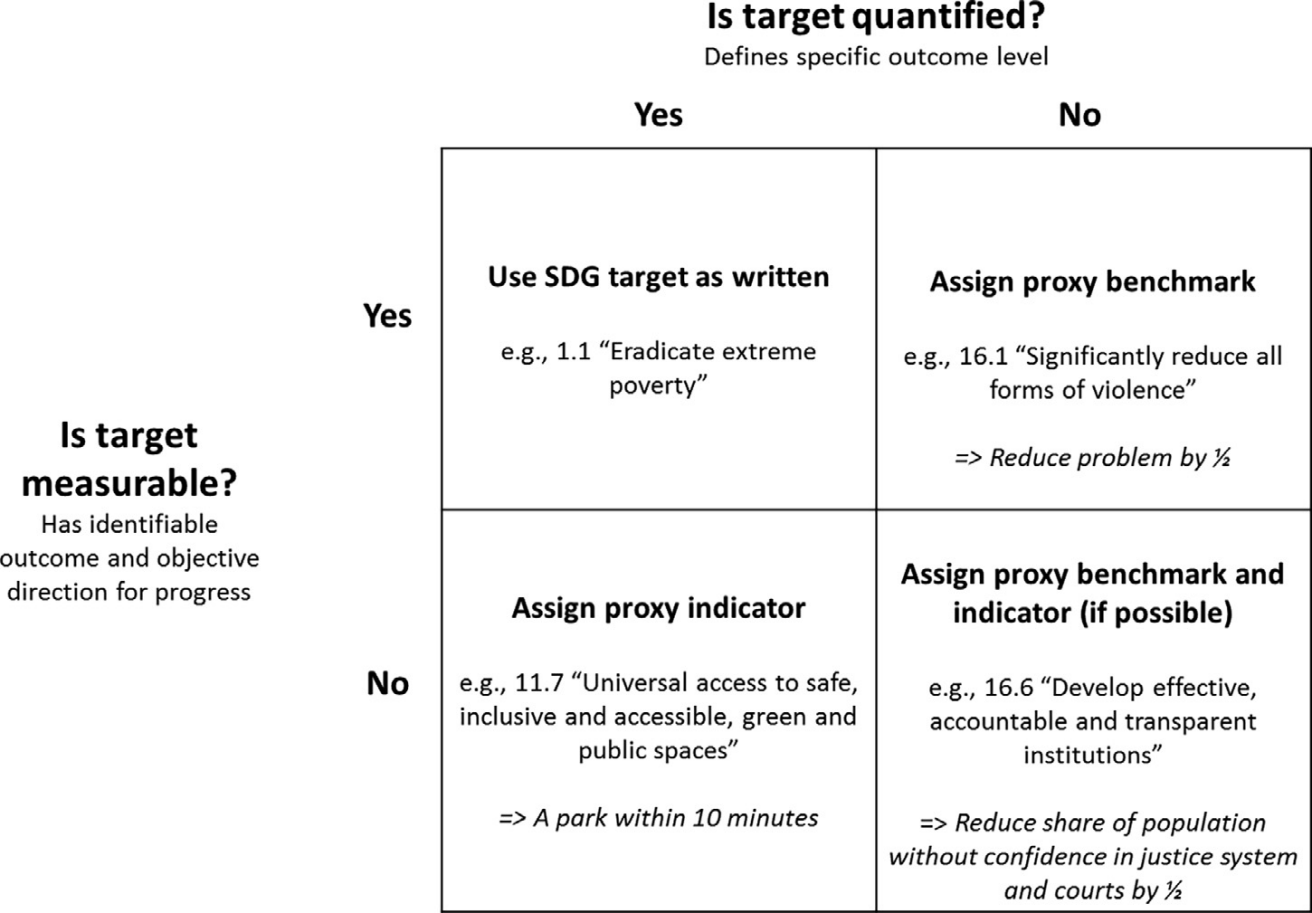
Logic for setting SDG proxy targets. Source: Authors.

In cases where we are not able to identify a corresponding national target, we propose a logic for establishing proxy targets, illustrated in Fig. 2. The horizontal axis segments targets by whether the official U.N. language defines a desired outcome level. The vertical axis segments by whether the official language includes a measurable outcome and objective direction of progress. As shown in the top-left quadrant, when targets are both measurable and quantified they are relatively straightforward to assess. In other cases, we attempt to assign a proxy benchmark, proxy indicator, or both, while recognizing the degree of subjectivity inherent in determining whether to interpret the wording of some targets as measurable or quantified. The Appendix shows our classification for each of the 59 relevant targets considered.

The top-right quadrant returns to the example of target 16.1 on violence, which is measureable but not quantified as written, and for which we can assign a proxy benchmark for assessing outcomes. As a general approach, we define benchmarks as cutting a relevant problem by half by 2030 – in this instance the intentional homicide rate. However, for targets that aim to increase a metric but do not have a natural data ceiling, such as target 9.5’s aim of, “substantially increasing the number of research and development workers per 1 million people,” we assign a 50 percent increase as the proxy benchmark. For targets under Goal 5 on gender equality, we use gender parity as a quantified benchmark for equality. A total of 17 targets fall under this top-right quadrant.

The bottom-left quadrant of Fig. 2 represents the cases where targets are quantified but not measurable, such as target 11.7’s aim to, “provide universal access to safe, inclusive and accessible, green and public spaces.” For seven such targets, we assign a proxy indicator, using official SDG indicators where practical. For example, target 11.7’s ambition of “universal access” to green and public spaces can be measured through a proxy indicator of the share of people living less than 10 min from a park or green space.

When a target is neither quantified nor measurable, as reflected in the bottom-right quadrant, the establishment of a proxy target is particularly subjective. In 19 instances, we are able to set a proxy target using a combination of the above approaches. Target 16.6, for example, is to “develop effective, accountable and transparent institutions.” In this instance we use a Statistics Canada indicator on public confidence in the justice system and courts and assign a proxy benchmark of halving the share of the population without confidence by 2030.^5^

For 16 targets, we deem it impractical to set a proxy because too much subjectivity is required. For example, target 12.2 is to, “achieve the sustainable management and efficient use of natural resources.” It is unclear what defines “efficient use” and the direction likely varies depending on which natural resource is considered, and in which context. Meanwhile, target 8.5 aims to, “achieve full and productive employment and decent work for all women and men.” In this case, the empirical standard for “full and productive employment” is unclear, especially in the context of debates about underemployment and “decent” wages.

**Fig. 3:**
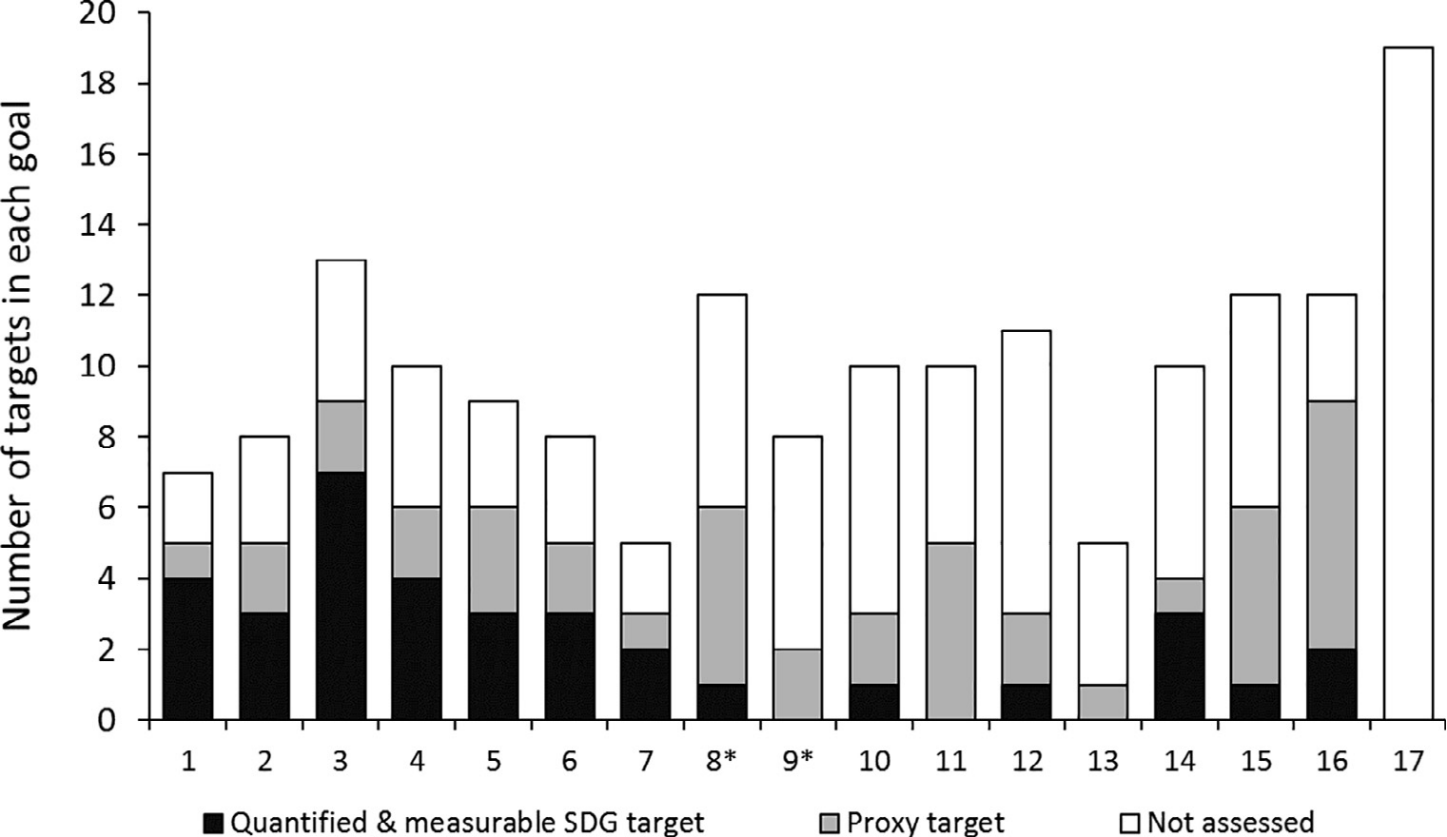
Assessable country-level SDG targets by goal. Note: * Numbers differ for the special case of least developed countries (LDCs). Targets 8.1 and 9.2 are only quantified and measurable at the country level for LDCs. For all other countries, these targets are considered not assessable. Source: Authors’ calculations.

Fig. 3 shows the spread of our target classifications across the 17 SDGs. On some goals, such as Goal 3 for health and wellbeing, we identify several directly quantified and measurable targets. For others goals, such as Goal 9 for industry, innovation, and infrastructure and Goal 11 for sustainable cities and communities, we are only able to make assessments through the use of proxy targets. Overall, the additional use of proxy targets results in a total of 78 quantitatively assessable outcome targets.^6^ The categorization of 35 quantified and measurable targets, 43 proxy targets, and 91 other forms of targets (here not assessed) applies generally across economies and levels of development. For least developed countries, the corresponding numbers are 37 quantified and measurable targets, 43 proxy targets, and 89 other forms of targets.

### 3.3 Identify data sources and indicators

**Fig. 4:**
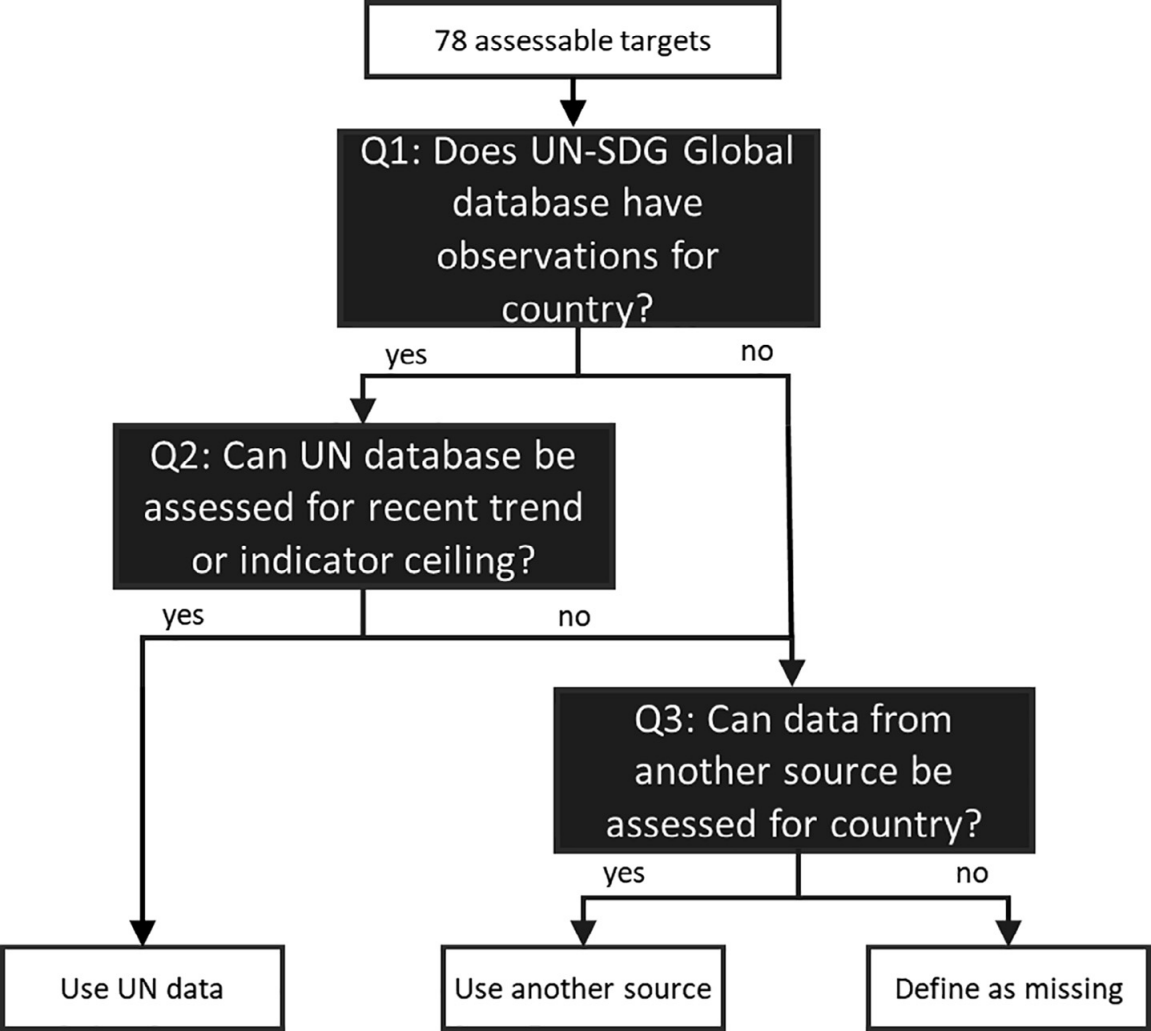
Logic for identifying SDG data sources. Source: Authors.

For the assessable targets, we identify relevant data sources using another decision tree, as outlined in Fig. 4. Here we first examine whether a target has any relevant data for the country of interest in the U.N. SDG Indicator Global Database, which we use as the default data source to allow for comparable analysis across countries. Because this database and others are updated regularly, Fig. 4 does not include a specific breakdown of how many targets fall under each branch of the tree. Next, we identify whether there are adequate observations to conduct recent trend analysis for the country, defined as either (i) having at least two observations since 2000, ideally spaced 10 years apart, or (ii) the most recent observation hitting an indicator ceiling that achieves the relevant target outcome (e.g., 100 percent access to basic drinking water). If the answer is no at either of the first two branches of the tree in Fig. 4, then the next step is to consider alternate sources, including the relevant national statistics agency. In doing so, we prioritize using indicators that match those in the SDG framework.

We aim to identify at least one indicator with data for each assessable target, although for nine targets we use two indicators to assess distinct outcomes that are embedded in the target language. For example, target 3.4 has two official indicators assessing two distinct issues: mortality from non-communicable diseases and suicide mortality rates. For targets with multiple official indicators, we prioritize using an indicator that assesses change in outcomes at the country-level and, where applicable, quantifies people being left behind. For example, on target 12.4 on sound management of chemicals and waste, we prioritize indicator 12.4.2 on hazardous waste generated per capita rather than 12.4.1 on parties to international agreements that meet their commitments and obligations in transmitting information.

### 3.4 Categorize each indicator’s 2030 trajectories

The fourth step in the methodology is to classify each indicator under a common analytical standard, based on its most recent trajectory. To do so, we first extrapolate each indicator’s recent trends, defined wherever possible as the ten-year period from 2007 to 2017, out to the SDG deadline.^7^ Following the logic of McArthur and Rasmussen (2018) we calculate proportional rates of progress (Eq. (1)) for mortality and economic growth indicators and absolute percentage point rates of progress (Eq. (2)) for all other indicators:

(1) $$\mathrm{Proportional}\;rate\;of\;progress={\left(\frac{x_{t}}{x_{t-n}}\right)}^{\frac{1}{n}}-1$$

(2) $$\mathrm{Percentage}\;point\;rate\;of\;progress=\frac{\left(x_{t}-x_{t-n}\right)}{n}$$

Here *x* represents the indicator value, *t* represents a recent index year, and *n* indicates the number of years prior to *t*, ideally 10 years.

We next extrapolate the recent trends over the remaining *y* years from *t* out to the target deadline, typically 2030, assuming an unchanged annual rate of progress, *r*. Eq. (3) shows the proportional trajectory calculation and Eq. (4) shows the percentage point trajectory:

(3) $$\mathrm{Proportional}\;trajectory={x_{t}\left(1+r\right)}^{y}$$

(4) $$\mathrm{Percentage}\;point\;trajectory=x_{t}+\left(r*y\right)$$

We then compare trajectories to each target’s desired outcomes, with particular attention to the SDG philosophy of “no one left behind.” As previously mentioned, for targets that commit to a desired outcome for “all” people or “universal” coverage, we interpret this literally as 100 percent of the population. Among other reasons, this is because each percentage point of population can represent a large number of lives. For example, if a country’s 2030 population is likely to be 50 million people, then every percentage point gap implies 500,000 people left behind. Even 98 percent population coverage on an indicator, which might generally be considered “success,” would still imply one million people left behind.

We classify each indicator trajectory under a common standard, using four categories to inform consideration of which issues are making progress and which ones are being left behind. Each category is based on the share of starting distance to the target that will be covered on current trajectory: (i) *On track*, meaning already achieved or on track for target achievement; (ii) *Acceleration needed*, meaning the country is currently on course to cover more than 50 percent but less than 100 percent of its starting distance to the target; (iii) *Breakthrough needed*, meaning the country is on course to cover between 0 and 50 percent of its starting distance to the target; and (iv) *Moving backwards*, meaning the most recent available trend is negative.^8^

### 3.5 Estimate the number of lives and people’s needs at stake

For indicators focused on human outcomes, the final step in the methodology is to translate trajectories into estimates of the absolute numbers of people left behind.^9^ We do so by calculating the approximate difference between the number of people affected on current trajectory and those affected under a trajectory that reaches the SDG target.^10^ For each indicator, calculations are based on the relevant demographic group, ranging from total population to narrower reference points like children aged 2 to 4 for children underweight, or females aged 15 and older for intimate partner violence.

For this portion of the analysis, we further segment targets into two conceptual categories: life-and-death targets like maternal mortality, traffic deaths, and homicides; and basic needs targets like food security, literacy, and access to water. For life and death targets, we estimate the cumulative number of “lives at stake” from a present year, here 2019, through to the target deadline, usually 2030. For basic needs targets, we estimate the number of people’s needs at stake in only the final target year, in order to avoid double counting.

### 3.6 Caveats

As with any analytical methodology, our approach has some inherent tradeoffs embedded. First, because we implement a literal interpretation of the SDG normative ambition to leave no one behind, our approach draws attention to shortfalls, however small, in reaching targets, rather than celebrating relative proximity to achieving an objective. For example, if access to some basic service is on course to climb from 99.4 percent in 2015 to 99.5 percent by 2030, then it is classified as a source of concern with a “breakthrough needed,” rather than an achievement, since less than half the remaining distance to the finish line of 100 percent would be covered. Similarly, if access to the same basic service had declined from 99.6 to 99.5 percent coverage in recent years, the target falls under the most problematic category of “moving backwards,” instead of something like “still close.”

Second, in instances where there are multiple options to choose from in selecting an indicator to assess a target, the choice of one indicator over another might provide different impressions of how a country is doing. For example, for target 3.3 on infectious diseases, we use the official indicator of tuberculosis incidence, due to its ongoing relevance across all countries. Using malaria incidence, another official indicator, might produce a different result, depending on its relevance to a particular country’s disease burden.

Third, targets anchored in relative domestic benchmarks risk conveying a negative narrative on indicators making considerable absolute gains but modest relative gains. To illustrate figuratively, if one indicator starts the SDG period 100 km from its target and only covers 40 km in 15 years, then this covers less than half the distance required and would be categorized under Breakthrough needed. Meanwhile, another indicator that starts the period 10 km away from its target and is on course to cover only 6 km, for a 60 percent gain, is categorized more positively as Acceleration needed.

Fourth, because we use a linear extrapolation for a number of trajectories (those not related to mortality or economic growth), recent fast-moving trends might overlook forthcoming “last mile” challenges en route to universal coverage and thereby risk overestimating current trajectories for 2030. Using a logistic function or similar adjustment would require inserting *ad hoc* assumptions regarding inflection points in basic needs trend lines, so we instead adhere to the straightforward calculations as described above.

## 4 Case study: applying the framework to Canada

To demonstrate the types of insights generated by our methodology, the following section applies the analytical framework to Canada. Although some readers might consider Canada to be a surprising case study for the issues, due to its higher values on many socioeconomic indicators than most low- and middle-income countries, it still grapples with many challenges of poverty and exclusion, most prominently among its indigenous peoples. The country has long fallen short, for example, in achieving universal access to basic drinking water.

In line with the intentionally universal nature of the intergovernmental policy agenda, many SDG targets are also set relative to the domestic nature of each country’s challenge, such as its own national poverty line, so a methodology needs to be able to accommodate cross-country variations in this regard. Moreover, there are some issues, like greenhouse gas emissions per capita and protection of coastal areas, on which Canada faces much bigger absolute challenges relative to many countries. Analytically, the country’s relatively good data availability also permits us to consider which types of insights can be generated through our methodology, which would be untestable in, for instance, an extremely resource-constrained country with no official statistics.

Implementing the methods described above, we identify relevant data for 61 outcome targets, using 70 indicators available as of March 2019. Twenty-eight of these indicators are drawn from the official U.N. SDG Indicator Global Database. Of these 28 indicators, Canada is missing trend data for two indicators but hits a relevant data ceiling. The other 42 indicators are drawn from complementary sources, including official Canadian government sources, as all described in the Appendix.

In considering potential data constraints to applying our framework to other countries, we note that Canada is not unique in at least some aspects of its data availability. It is beyond the scope of this study to look at all potential domestic data sources for all countries, but if one looks at the Group of 20 countries as a relevant cross-section, then of the 26 indicators for which Canada has trend data available in the U.N. SDG Indicator Global Database as of March 2019, all G-20 countries have relevant trend data for at least 20 indicators, and four countries – Argentina, Italy, Mexico, and Turkey – have trend data for the same 26 indicators. Some developing G-20 countries also have more indicators available in the U.N. database than Canada does.

### 4.1 Which issues are getting left behind?

As our first main empirical result, Table 1 presents a goal-by-goal summary classification of all 70 indicators examined for Canada. Solid circles represent indicators for targets that are quantified and directly measurable as written. Hollow circles represent indicators for targets that are assessed by a proxy measure. The Appendix provides each indicator’s underlying numerical values and corresponding classification.

**Table 1:** Case study: Summary of Canada’s status on domestic SDG indicators.

	Sustainable Development Goal	Moving backwards	Breakthrough needed	Acceleration needed	On track
1	Poverty				●●●○
2	Hunger & food systems	●●○	●		
3	Good health & well-being		●●●●	●●○	●●○
4	Quality education	●	●●		●●●
5	Gender equality		●●●○○○		
6	Clean water & sanitation	●●	●○○		○
7	Affordable & clean energy	○	●	○	●
8	Decent work & economic growth		○○○		○
9	Industry, innovation & infrastructure	○	○○		
10	Reduced inequalities			○	●
11	Sustainable cities & communities	○	○○		○
12	Responsible consumption & production		●○○		
13	Climate action		○		
14	Life below water			●●	●
15	Life on land		○○○		○
16	Peace, justice & strong institutions	●○○	○○		●

	Total	12	33	7	18

● Denotes indicator for SDG target that is quantified and directly measurable as written.

○ Denotes indicator for SDG target assessed by proxy measure.

Source: Authors’ calculations using Centre for Research on the Epidemiology of Disasters, 2017, Cotter, 2015, Environment and Climate Change Canada, 2018a, Environment and Climate Change Canada, 2018b, Environment and Climate Change Canada, 2018c, Environment and Climate Change Canada, 2018d, Environment and Climate Change Canada, 2019a, Environment and Climate Change Canada, 2019b, Environment and Climate Change Canada, 2019c, Environment and Climate Change Canada, 2019d, Global Burden of Disease Collaborative Network, 2018, Gooch et al., 2019, Kaufmann and Kraay, 2018, National Energy Board, 2017, Natural Resources Canada, 2018, OECD, 2019a, OECD, 2019b, OECD., 2019c, OECD, 2019d, OECD, 2019e, Public Safety Canada, 2019, Roberts, 2004, Statistics Canada, 2013, Statistics Canada, 2017, Statistics Canada, 2019a, Statistics Canada, 2019b, Statistics Canada, 2019c, Statistics Canada, 2019d, Statistics Canada, 2019e, Statistics Canada, 2019f, Statistics Canada, 2019g, Statistics Canada, 2019h, Statistics Canada, 2019i, UNESCO, 2017, United Nations Statistics Division, 2019, World Bank, 2019, World Data Lab, 2019.

Overall, the table suggests that Canada is so far only fully on track for one of the first 16 SDGs, Goal 1 on ending poverty. For Goals 2 through 16, the country requires faster progress on at least one indicator, even if for many indicators the absolute distance to the SDG benchmark is small. In total, the country is on track for 18 indicators; requires acceleration on 7; needs a clear breakthrough on progress on 33; and requires a reversal of trends on 12. While Canadian society has undoubtedly achieved success on many fronts, efforts are still needed to cover a “last mile” of success on many issues and to achieve faster overall progress on others.

The “On track” column on the right of Table 1 shows the positive aspects of the results. Before the establishment of the SDGs in 2015, Canada had already surpassed absolute global standards for targets including extreme income poverty (under Goal 1), neonatal mortality (Goal 3), and maternal mortality (also Goal 3), and had achieved universal access to services like social protection (under Goal 1), modern energy (Goal 7), and legal identity (Goal 16). Canada’s current trajectory also places it on track to meet multiple targets including halving the share of domestic poverty (under Goal 1), and achieving universal access to early childhood education and universal upper secondary graduation rates (under Goal 4) by 2030. Issues falling under this column suggest related policy approaches have generally been working well.

The “Acceleration needed” column captures indicators that are making good but not quite enough progress to achieve the targets. For example, Canada requires acceleration to meet targets on preventing marine pollution (under Goal 14) and increasing renewable energy generation (under Goal 7). These are issues where targeted efforts might be needed to “nudge” efforts toward greater success (Sunstein and Thaler, 2008, Biggs and McArthur, 2018).

At the other end of the spectrum, the far-left column of Table 1 draws attention to national challenges where indicators have been moving backwards. Under Goal 2, for example, indicators of food insecurity and children overweight have been worsening. Remarkably, reported access to drinking water (under Goal 6) has recently declined, the problem being particularly concentrated among indigenous people. Under Goal 4 on education, the proportion of lower secondary students who lack basic numeracy skills has been increasing.

The “Breakthrough needed” column reflects indicators that are either stagnant or making slow progress toward the targets. National breakthroughs are needed for Canada to achieve gender equality, Goal 5, as measured by the wage gap, gender disparity in unpaid work, violence against women, early marriage, and women in managerial positions. For indicators falling under either the Breakthrough needed column or the Moving backwards column, recent policy approaches appear not to have been working well enough, so new strategies are likely required.

In addition to reviewing results by column category, our methodology enables mapping of diverse issue-specific dynamics within each goal domain. For example, the range of issues encompassed in Goal 3 draws attention to Canada’s mixed trajectories for health and well-being. Mortality from non-communicable diseases is declining, although needs acceleration in order to achieve a one-third reduction by 2030. Faster progress is also needed to reduce substance abuse and to cut, by 2020, traffic deaths by half. The country is nearly but not quite on track to achieve universal coverage of nine key health interventions by 2030. A breakthrough is required on suicide mortality, infectious diseases like TB, and universal access to reproductive health services.

Meanwhile, Canada’s outlook on environmental issues is mixed. The federal government has recently established, and made concrete steps toward meeting, SDG-consistent targets for protecting its uncommonly large land and marine areas by 2020, although faster progress is still needed to achieve desired outcomes on both fronts. Meanwhile, the country seems to be on course to end overfishing and keep forest harvests within sustainable levels, but a breakthrough is required to halt the loss of biodiversity. On climate change, Canada has some of the world’s highest per capita emissions and requires breakthrough rates of progress to meet its own 2030 emissions targets, again despite recent policy advances. Relevant indicators also show the need to increase energy efficiency and the share of renewables in energy consumption.

In light of Canada’s international reputation for good governance, the results under Goal 16 for peace, justice, and strong institutions offer potential surprise. Many aspects of the country’s public institutions are strong, but only 57 percent of the population has clear confidence in the justice system and courts. Indicators are moving in the wrong direction for reported sexual violations against children and unsentenced detainees as a share of the prison population. Future research focused on Canada’s domestic Goal 16 challenges would clearly be valuable.

Our methodology also permits a deeper dive on such issues at the subnational level. As an illustration, Table 2 maps status on one component of SDG target 3.4, major cardiovascular disease mortality, for each of Canada’s ten provinces and three territories. Whereas the country as a whole needs acceleration to achieve the overall NCD mortality target, it is on track to achieve a one-third reduction in the cardiovascular disease mortality component. Unpacking the national trend by geography, fully seven provinces and two territories are currently off track. The results suggest that the Northwest Territories and Nunavut merit particular attention due to slow and even backwards rates of progress. These two territories each have populations of less than 50,000 people, but more than half of each population is comprised of indigenous people, drawing attention to Canada’s unique historical challenge in supporting relevant communities. Further national disaggregation by location, gender, indigenous status, income group, age, immigrant status, and disability status can reveal similar insights wherever data permit.

**Table 2:** Summary of status on major cardiovascular disease mortality by Canadian province and territory (age-standardized per 100,000 people).

Province or territory	Moving backwards	Breakthrough needed	Acceleration needed	On track
Alberta			●	
British Columbia			●	
Manitoba			●	
New Brunswick			●	
Newfoundland and Labrador				●
Nova Scotia			●	
Ontario				●
Prince Edward Island			●	
Quebec				●
Saskatchewan			●	
Northwest Territories		●		
Nunavut	●			
Yukon				●

Total	1	1	7	4

Note: ● indicates the trajectory classification for cardiovascular disease mortality in the province or territory.

Source: Authors’ calculations using Statistics Canada, 2019d.

As an empirical reference point, our national results for Canada can be compared to those produced by SDSN (Sachs et al., 2018). Some findings are a naturally direct match. For example, on targets 3.1 for maternal mortality and 3.2 for neonatal mortality, both we and SDSN identify Canada as already meeting the absolute thresholds identified in the targets. On target 6.2 for access to safely managed sanitation, SDSN classifies Canada as “Stagnating” or “increasing at a rate below 50% of the growth rate needed to achieve the SDGs,” which is similar to our classification of Breakthrough needed.

Meanwhile, one important source of difference between our results and those of SDSN is our literal interpretation of “no one left behind” for absolute targets. As mentioned earlier, SDSN uses a lower threshold for targets aiming at universal coverage. For example, under target 6.1 for access to drinking water, SDSN classifies high-income and OECD countries as achieving the SDG if they have at least 95 percent of the population using safely managed water services and classifies all other countries as achieving if they have 98 percent or more using at least basic water services. While SDSN does not classify a drinking water trend for Canada, World Bank (2019) data reports Canada’s 2015 value for basic water services as above the SDSN threshold but short of universal access and moving backwards. We interpret the SDG target to require fully 100 percent coverage and our approach draws attention to both the gap and the trajectory.^11^ Recognizing the importance of real-time measurement for this issue in Canada and other contexts, in light of the large amount of Canadian public attention focused on shortfalls in drinking water access for many of the country’s indigenous communities, we believe our methodology aligns well with the “no one left behind” intention that underpins the SDGs.

A second key source of difference in results is anchored in our by-the-book treatment of relative targets as proportional objectives for each country, rather than common absolute objectives across countries. To illustrate again with target 3.4 on NCDs, as mentioned earlier, SDSN defines SDG achievement as meeting an absolute global mortality threshold, which Canada has already surpassed, whereas our method sets Canada’s benchmark relative to its own initial baseline. Our calculations indicate that Canada is not yet on course to reduce its NCD mortality rate by one-third by 2030, and we therefore classify the relevant indicator as Acceleration needed.^12^

### 4.2 How many people’s lives and basic needs are at stake?

The findings produced by our methodology do not amount to predictions, nor a suggestion that an assessed country can or cannot meet the SDGs. Instead, the results draw attention to a country’s gaps: the issues and people that are currently being left behind amid a society’s pursuit of economic, social, and environmental progress. We next translate the gaps on a subset of outcome trajectories into their absolute human consequences. We stress that these findings are only approximate estimates, meant to demonstrate a transparent quantitative methodology by which analysts and policymakers could consider the people-focused implications of intergovernmental commitments.

To that end, Table 3 presents the estimated number of people left behind for a cross-section of targets that are measurable and for which Canada is not currently on track. The right-side column indicates the relevant reference population. The 2017 U.N. population projection suggests that, by 2030, Canada will have a total population of 40.6 million people, up from 37.6 million in 2020, the deadline year for the traffic death target. Across life and death targets, we estimate that current shortfalls translate to more than 54,000 Canadian lives at stake between 2019 and 2030. This includes 44,000 lives lost to gaps in reducing premature mortality from non-communicable diseases. On suicide, the shortfall translates to 8000 lives at stake. On traffic deaths, Canada has been making progress toward the 2020 target deadline, but shortfalls in cutting mortality by half will amount to 900 additional lives lost in 2019 and 2020. Meanwhile the homicide rate has seen little progress, and the shortfall toward achieving a proxy benchmark of 50 percent reduction by 2030 translates to an additional 2000 deaths.

**Table 3:** Estimating SDG target gaps measured by lives at stake in Canada.

	BAU value in 2030	Value required to meet target	Lives at stake, 2019–30^†^	Reference population in 2030
*Directly measured targets*				
Mortality rate due to non-communicable diseases (aged 30–70, per 100,000)	172	142	44,000	21,119,000
Suicide mortality rate, per 100,000	11.4	8.3	8,000	40,618,000
Death rate due to road injuries, per 100,000	4.5^*^	3.0^*^	900^*^	37,603,000^*^

*Proxy target*				
Rate of homicide, per 100,000	1.5	0.8	2,000	40,618,000

**Total**			**54,900**	

Notes: ^†^Lives at stake estimates are rounded to the nearest thousand. If total is less than one-thousand, numbers are rounded to nearest hundred. * Indicates target end year is 2020 and reference population is for 2020. Trajectory values are based on methodology described in paper. Traffic death mortality is estimated as cumulative for 2019 and 2020. Mortality estimates for non-communicable diseases, suicide, and homicide are cumulative from 2019 to 2030. Source: Authors' calculations using Global Burden of Disease Collaborative Network, 2018, United Nations Population Division, Department of Economic and Social Affairs (U.N.-DESA), 2017, United Nations Statistics Division, 2019.

For measures of basic needs in Table 4, we estimate the number of people’s needs at stake in the final target year, if trajectories fall short of the desired outcome, again separating out results for proxy targets.^13^ Unlike for lives at stake, the numbers in Table 4 are not strictly comparable from one row to the next because indicators are measured relative to different reference populations, as again listed in the final column. Access to water, for example, is measured relative to the entire population of Canada, whereas children overweight is reported only for children aged 2–4.

**Table 4:** Estimating SDG basic needs target gaps measured by people left behind in Canada.

	BAU value in 2030	Value required to meet target	People left behind in 2030^†^	Reference population in 2030
*Directly measured targets*				
Moderate & Severe food insecurity (applied to total population)	11.0%	0%	4,448,000	40,618,000
Children overweight, (aged 2–4)	29.2%	0%	356,000	1,218,000
TB incidence, per 100,000	4.9	1	12,000	40,618,000
Women with family planning needs satisfied (aged 15–49)	87.5%	100%	1,089,000	8,724,000
Minimum proficiency in reading, lower secondary (applied to population aged 15–79)	89.8%	100%	3,244,000	31,734,000
Minimum proficiency in mathematics, lower secondary (applied to population aged 15–79)	79.7%	100%	6,449,000	31,734,000
Women experiencing intimate partner violence (aged 15–79, age-standardized)	5.7%	0%	901,000	15,893,000
Police-reported female victims of violent crime, per 100,000 females aged 0–79	685	0	130,000	18,946,000
Share of 15–17 year old females who are married	0.034%	0%	200	655,000
Access to basic drinking water services	97.4%	100%	1,056,000	40,618,000
Access to safely managed sanitation services	80.4%	100%	7,966,000	40,618,000

*Proxy targets*				
Women in managerial positions (applied to females aged 0–79)	36%	50%	5,453,000	18,946,000
Youth not in education, employment or training (aged 15–24)	10.2%^*^	5.1%^*^	215,000^*^	4,262,000^*^
Have park or green space <10 min from home	91%	100%	3,757,000	40,618,000
Confidence in institutions - Justice system and courts (great deal or some)	57%	78%	8,733,000	40,618,000

Notes: **^†^**Estimated numbers of people left behind are rounded to the nearest thousand. If total is less than one-thousand, numbers are rounded to nearest hundred. * Indicates target end year is 2020 and reference population is for 2020. TB incidence is cumulative from 2019 to 2030.

Trajectory values are based on methodology described in paper. Food insecurity estimates apply indicator measuring share of people aged 12 and older to entire population. Proficiency in reading and mathematics applies indicator measuring share of those in lower secondary to the population 15 and older. We interpret women in managerial positions as a general proxy for gender discrimination and apply indicator to the total female population.

Source: Authors' calculations using Global Burden of Disease Collaborative Network, 2018, Roberts, 2004, Cotter, 2015, Statistics Canada, 2019c, Statistics Canada, 2019e, Statistics Canada, 2019f, Statistics Canada, 2019h, United Nations Population Division, Department of Economic and Social Affairs (U.N.-DESA), 2017, United Nations Statistics Division, 2019, World Bank, 2019.

The results in Table 4 show that, although Canada is often close in percentage point terms to achieving many of the SDG targets, the shortfalls frequently translate to millions of people left behind. The extrapolation of recent trends implies more than one million people without access to basic drinking water by 2030 and almost eight million without safely managed sanitation services. On education, approximately 3.2 million Canadians aged 15–79 might lack core literacy skills by the same year, while more than 6.4 million might lack core numeracy skills. As an indicator for local quality of life, more than 3.7 million people would not have access to a park or green space within ten minutes of their home.

If hunger trends continue, more than 4.4 million people in Canada will suffer from moderate or severe food insecurity in 2030. Concurrently, more than 29 percent of children aged 2–4 are on trend to be overweight by the same year. This is equivalent to 356,000 children and implies much greater overall numbers of Canadians of all ages struggling with overweight or obese status over the coming decade.

The results also draw attention to the number of Canadian women and girls who will be left behind if recent trends continue. As a general proxy for gender discrimination, we interpret the share of women in managerial positions as representative of overall barriers to women’s equal access to leadership positions in society. On current trajectory, only 36 percent of managers in Canada will be women in 2030, far short of half. Extrapolating this gap across society implies 5.5 million Canadian women and girls being excluded from equal opportunities. On the issue of intimate partner violence, the shortfall to elimination translates to more than 900,000 women in 2030.

## 5 Conclusion

This paper began by asking how useful the SDGs are in informing country-level empirical analysis across economic, social, and environmental indicators. We pursue this question with an aim of identifying a quantitative methodology for assessing which people and issues are being left behind. We provide a tractable analytical framework for generating answers to these questions, adhering as much as practical to the formal U.N. targets, indicators, and database. Our results can inform the ongoing evolution of methodological debates regarding best approaches to SDG measurement.

Of the 169 SDG targets, we find that many, although not a majority, are useful for empirical assessment. Specifically, we classify 35 targets as directly outcome-focused, quantified, and measurable at the national level for all countries. Another two targets meet the same criteria for LDCs. We identify another 43 targets that are plausibly assessable across all countries through the use of “proxy” benchmarks and indicators. This yields a total of 78 assessable SDG outcome targets at the country level. That slightly less than half the SDG targets are assessable at the country level forms something of a Rorschach test: some readers might see this as a cup half full of assessable targets; others might see it as a cup half empty of missed opportunities.

As a case study, we apply our methodology to Canada. In attempting to identify data sources to inform trajectory analysis, we are only able to identify relevant data for 61 targets, using 70 indicators, of which 28 indicators can be directly sourced from the U.N. SDG Indicator Global Database. Although it might be reasonable to presume that Canada is close to a global upper bound in terms of SDG data availability, its indicators drawn from the U.N. database have similar availability across G-20 countries. But it is again a matter of interpretation as to whether 61 targets and 70 indicators amount to large or small numbers in the SDG context. They represent less than a third of all SDG targets and indicators but still add up to several dozen measures of progress.

We apply a classification scheme to harmonize and distill trajectory diagnoses across all indicators, including those aiming at absolute global targets and those aimed at domestically-referenced relative targets. We classify each indicator under one of four categories – On track, Acceleration needed, Breakthrough needed, or Moving backwards – based on the share of the initial target gap that the country is on course to address by the relevant SDG deadline. The categorization of indicators can help inform policy making. Issues that are on track likely merit the continuation of recent approaches. Those with acceleration needed might need a policy tweak or targeted effort to close the remaining gap. Indicators that are relatively stagnant and register under breakthrough needed likely merit a change in strategy. Indicators that are moving backwards require a turnaround strategy, potentially requiring a wholesale new approach from all relevant stakeholders.

In the Canadian context, we find 18 indicators to be on track for success, 7 requiring acceleration, 33 requiring a breakthrough, and 12 moving in the wrong direction. Overall, looking across our assessed indicators, Canada is only wholly on track for one of the first 16 SDGs: Goal 1 on poverty. Despite the country’s many notable successes, it exhibits a pattern of issues and population segments being consistently left behind. This type of trajectory analysis could help researchers and policy leaders identify priority areas for focusing attention on the need for change.

The final step of the framework is to estimate the human consequences of SDG shortfalls if recent trends continue. In the Canadian case study, our results suggest that more than 54,000 lives are at stake if trajectories continue to fall short of SDG targets. For measures of basic needs at stake relative to SDG achievement, shortfalls might imply more than 4.4 million people subject to food insecurity, more than 6.4 million adults lacking core numeracy skills, and more than 900,000 women subject to intimate partner violence. These estimates should not be interpreted with false precision, since they are based on a number of methodological assumptions in extrapolating recent trends. But the results do offer a practical approach to translating abstract statistical percentages in to specific numbers of people.

We show how corresponding analysis can be conducted for indicators at the subnational level. In Canada, there are tremendous variations across geographies and demographic groups. Among the considerable populations currently being left behind, the SDGs draw particular attention to the profound and longstanding challenges still faced by indigenous people in Canada.

Our methodology is not able to assess status on all SDG-relevant issues, often because the relevant target is not defined in a clear or relevant manner. For example, the targets on industry and infrastructure are not ideal for assessing performance in an advanced economy, nor are many of the targets on decent work and economic growth. Some countries might want to set their own SDG targets in these realms. Future research might also consider more refined approaches to establishing proxy targets.

Overall, this study shows that, despite data gaps and limitations, a subset of assessable and outcome-focused SDG targets offer a useful framework for conducting an empirical diagnosis of country-level economic, social, and environmental trends. The relevance for empirical diagnosis in a high-income country like Canada suggests a more universal applicability across countries grappling with lower general levels of development and greater intensities of human deprivation. Ideally, a quantitative distillation of diplomatically-defined challenges can then inform necessary policy debates on how best to solve problems if, in fact, people and issues are not to be left behind.

## Declaration of Competing Interest

The authors have no conflict of interest in preparing this paper.

## Footnotes

1 A limited number of targets have 2020 or 2025 deadlines. See Appendix for relevant details.

2 We note that target 14.1, to “prevent and significantly reduce marine pollution of all kinds,” could be interpreted as either an absolute or relative target. For the purposes of this paper we interpret “prevent” as an absolute threshold of zero marine pollution spills.

3 Target 8.1 aims to, “Sustain […] at least 7 percent gross domestic product growth per annum in the least developed countries” and target 9.2 aims to “double [industry’s] share in least developed countries.”

4 In the Canadian context, we identify six national targets that can serve as proxy targets. For all six corresponding SDG targets, if no national target were available then we would still be able to establish proxy targets through the logic described in this section.

5 Specifically, we draw from Cotter, 2015, which reports a 2013 Statistics Canada survey question on the share of people with “a great deal of confidence” and “some confidence” in the justice system and courts, alongside categories of “neutral,” “not very much confidence,” and “no confidence at all.” To estimate underlying trends, we also draw from Roberts, 2004, which reports a 2003 Statistics Canada survey question on the share of people with “a great deal of confidence” and “quite a lot of confidence” in the justice system, alongside “not very much confidence” and “no confidence at all.” The imperfect nature of such trend approximations only underscores the importance of generating proper time-series data to assess relevant SDG targets.

6 Again, this total of 78 targets holds even if no corresponding national targets exist.

7 In some instances, reference periods are adjusted due to data limitations. For seven indicators with considerable year-to-year volatility within Canada (under targets 1.2, 2.4, 5.5, 8.6, 14.1, 16.1, 16.5), we calculate a linear fit using available observations from 2005 to 2017. See Appendix for indicator-specific calculations.

8 For two targets, we use only three classification categories. For target 6.6 on protecting and restoring water-related ecosystems, we classify trajectories as “On track” if they exceed initial ecosystem size, “Breakthrough needed” if they have no change, and “Moving backwards” if they decline. For target 10.1 on income growth of the bottom 40 percent compared to national averages, we classify trajectories as On track if growth rates exceed the national average, Breakthrough needed if they are equal, and Moving backwards if they are slower.

9 By focusing on indicators measured in terms of people, we include, for example, the share of people with access to electricity (target 7.1) but not the share of renewable energy consumed (target 7.2).

10 This builds on methods previously described in McArthur et al. (2018) and Kharas et al. (2018).

11 In comparing results, we note that the SDSN index methodology is updated from year to year, often incorporating feedback from external researchers such as ourselves.

12 We also use a slightly different NCD indicator (age-standardized mortality rate in populations aged 30–70 per 100,000) than SDSN, but indicator choice is not the main driver of difference in results.

13 The exception is for tuberculosis, on which we calculate cumulative new infections from 2019 through 2030.

## References

[b0005] Biggs M., McArthur J.W., Desai R.M., Kato H., Kharas H., McArthur J.W. (2018). A Canadian North Star: Crafting an advanced economy approach to the Sustainable Development Goals. From summits to solutions: Innovations in implementing the Sustainable Development Goals.

[b0010] Centre for Research on the Epidemiology of Disasters. (2017). Emergency events database (EM-DAT) [Data file]. Retrieved from http://www.emdat.be/ (accessed May 8, 2017).

[b0015] Cotter A. (2015). Public confidence in Canadian institution. Statistics Canada catalogue no. 89- 652-x2015007. Spotlight on Canadians: Results from the General Social Survey.

[b0020] Cross Sectoral Coordination Centre of Latvia (2018). Latvia: Implementation of the Sustainable Development Goals.

[b0025] Cuaresma J.C., Fengler W., Kharas H., Bekhtiar K., Brottrager M., Hofer M. (2018). Will the Sustainable Development Goals be fulfilled? Assessing present and future global poverty. Palgrave Communications.

[b0030] Environment and Climate Change Canada (2016). Achieving a sustainable future: A Federal Sustainable Development Strategy for Canada 2016–2019.

[b0035] Environment and Climate Change Canada. (2018a). Canada's conserved areas [Data file]. Retrieved from https://www.canada.ca/en/environment-climate-change/services/environmental-indicators/conserved-areas.html (accessed March 17, 2019).

[b0040] Environment and Climate Change Canada. (2018b). Marine pollution spills [Data file]. Retrieved from https://www.canada.ca/en/environment-climate-change/services/environmental-indicators/marine-pollution-spills.html (accessed March 17, 2019).

[b0045] Environment and Climate Change Canada. (2018c). Solid waste diversion and disposal [Data file]. Retrieved from https://www.canada.ca/en/environment-climate-change/services/environmental-indicators/solid-waste-diversion-disposal.html (accessed March 17, 2019).

[b0050] Environment and Climate Change Canada. (2018d). Sustainable fish harvest [Data file]. Retrieved from https://www.canada.ca/en/environment-climate-change/services/environmental-indicators/sustainable-fish-harvest.html (accessed March 17, 2019).

[b0055] Environment and Climate Change Canada. (2019a). Air quality [Data file]. Retrieved from https://www.canada.ca/en/environment-climate-change/services/environmental-indicators/air-quality.html (accessed March 17, 2019).

[b0060] Environment and Climate Change Canada. (2019b). Progress towards Canada's greenhouse gas emissions reduction target [Data file]. Retrieved from https://www.canada.ca/en/environment-climate-change/services/environmental-indicators/progress-towards-canada-greenhouse-gas-emissions-reduction-target.html (accessed March 17, 2019).

[b0065] Environment and Climate Change Canada. (2019c). Species at risk population trends [Data file]. Retrieved from https://www.canada.ca/en/environment-climate-change/services/environmental-indicators/species-risk-population-trends.html (accessed March 17, 2019).

[b0070] Environment and Climate Change Canada. (2019d). Water quality in Canadian rivers [Data file]. Retrieved from https://www.canada.ca/en/environment-climate-change/services/environmental-indicators/water-quality-canadian-rivers.html (accessed March 17, 2019).

[b0075] European Union (EU) (2017). Sustainable development in the European Union: Monitoring report on progress towards the SDGs in an EU context 2017 edition.

[b0080] Global Burden of Disease 2017 Collaborators (2018). Measuring progress from 1990 to 2017 and projecting attainment to 2030 of the health-related Sustainable Development Goals for 195 countries and territories: A systematic analysis for the Global Burden of Disease Study 2017. The Lancet.

[b0085] Global Burden of Disease Collaborative Network. (2018). Global Burden of Disease Study 2017 (GBD 2017) Health-related Sustainable Development Goals (SDG) Indicators 1990-2030 [Data file]. Retrieved from http://ghdx.healthdata.org/record/global-burden-disease-study-2017-gbd-2017-health-related-sustainable-development-goals-sdg (accessed March 16, 2019).

[b0090] Gooch M., Bucknell D., LaPlain D., Dent B., Whitehead P., Felfel A., Maguire M. (2019). The avoidable crisis of food waste: Technical report.

[b0095] Kaufmann, D. & Kraay, A. (2018). The Worldwide Governance Indicators, 2018 Update [Data file]. Retrieved from http://info.worldbank.org/governance/wgi/ (accessed March 17, 2019).

[b0100] Kharas H., McArthur J.W., Rasmussen K. (2018). How many people will the world leave behind?. Brookings Global Economy and Development Working Paper No. 123.

[b0105] Kindornay S. (2019). Progressing national SDG implementation: An independent assessment of the voluntary national review reports submitted to the United Nations High-Level Political Forum in 2018.

[b0110] McArthur J.W., Rasmussen K. (2018). Change of pace: Advances and accelerations during the Millennium Development Goal era. World Development.

[b0115] McArthur J.W., Rasmussen K., Yamey G. (2018). How many lives at stake? Assessing 2030 Sustainable Development Goal trajectories for maternal and child health. The BMJ.

[b0120] Ministry of Planning, Monitoring, and Administrative Reform (2018). Egypt’s voluntary national review 2018.

[b0125] National Energy Board (2017). Canada's renewable power landscape: Energy market analysis 2017.

[b0130] Natural Resources Canada. (2018). Indicator: Volume harvested relative to the sustainable wood supply [Data file]. Retrieved from https://www.nrcan.gc.ca/forests/report/harvesting/16550 (accessed March 17, 2019).

[b0135] Nicolai S., Hoy C., Berliner T., Aedy T. (2015). Projecting progress: Reaching the SDGs by 2030.

[b0140] Organisation for Economic Cooperation and Development (2017). Measuring distance to the SDG targets: An assessment of where OECD countries stand.

[b0145] Organisation for Economic Cooperation and Development. (2019a). Gender wage gap [Data file]. Retrieved from https://data.oecd.org/earnwage/gender-wage-gap.htm (accessed March 17, 2019).

[b0150] Organisation for Economic Cooperation and Development. (2019b). Nutrient balance [Data file]. Retrieved from https://data.oecd.org/agrland/nutrient-balance.htm (accessed March 17, 2019).

[b0155] Organisation for Economic Cooperation and Development. (2019c). OECD.Stat: Income distribution and poverty [Data file]. Retrieved from https://stats.oecd.org/Index.aspx?DataSetCode=IDD (accessed March 17, 2019).

[b0160] Organisation for Economic Cooperation and Development. (2019d). Secondary graduation rate [Data file]. Retrieved from https://data.oecd.org/students/secondary-graduation-rate.htm (accessed March 17, 2019).

[b0165] Organisation for Economic Cooperation and Development. (2019e). Waste water treatment [Data file]. Retrieved from https://data.oecd.org/water/waste-water-treatment.htm (accessed March 17, 2019).

[b0170] Public Safety Canada. (2019). Canadian Disaster Database [Data file]. Retrieved from https://www.publicsafety.gc.ca/cnt/rsrcs/cndn-dsstr-dtbs/index-en.aspx (accessed March 17, 2019).

[b0175] Roberts J.V. (2004). Public confidence in criminal justice: A review of recent trends 2004–05. Report for Public Safety and Emergency Preparedness Canada.

[b0180] Sachs J., Schmidt-Traub G., Kroll C., Lafortune G., Fuller G. (2018). SDG index and dashboards report 2018.

[b0185] Statistics Canada (2013). Homeownership and shelter costs in Canada. Analytical document using National Household Survey, 2011.

[b0190] Statistics Canada (2017). Housing in Canada: Key results from the 2016 Census. The Daily.

[b0195] Statistics Canada. (2019a). Table 11-10-0134-01 – Gini coefficients of adjusted market, total and after-tax income [Data file]. Retrieved from https://www150.statcan.gc.ca/t1/tbl1/en/cv.action?pid=1110013401 (accessed March 16, 2019).

[b0200] Statistics Canada. (2019b). Table 11-10-0135-01 – Low income statistics by age, sex and economic family type [Data file]. Retrieved from https://www150.statcan.gc.ca/t1/tbl1/en/tv.action?pid=1110013501 (accessed March 16, 2019).

[b0205] Statistics Canada. (2019c). Table 13-10-0463-01 - Household food insecurity, by age group and food insecurity status [Data file]. Retrieved from https://www150.statcan.gc.ca/t1/tbl1/en/tv.action?pid=1310046301 (accessed March 16, 2019).

[b0210] Statistics Canada. (2019d). Table 13-10-0800-01 – Deaths and mortality rate (age standardization using 2011 population), by selected grouped causes [Data file]. Retrieved from https://www150.statcan.gc.ca/t1/tbl1/en/tv.action?pid=1310080001 (accessed March 16, 2019).

[b0215] Statistics Canada. (2019e). Table 17-10-0060-01 – Estimates of population as of July 1st, by marital status or legal marital status, age and sex [Data file]. Retrieved from https://www150.statcan.gc.ca/t1/tbl1/en/cv.action?pid=1710006001 (accessed March 16, 2019).

[b0220] Statistics Canada. (2019f). Table 35-10-0051-01 – Victims of police-reported violent crime and traffic offences causing bodily harm or death, by type of offence and sex and age group of victim [Data file]. Retrieved from https://www150.statcan.gc.ca/t1/tbl1/en/tv.action?pid=3510005101 (accessed March 18, 2019).

[b0225] Statistics Canada. (2019g). Table 35-10-0177-01 – Incident-based crime statistics, by detailed violations [Data file]. Retrieved from https://www150.statcan.gc.ca/t1/tbl1/en/cv.action?pid=3510017701 (accessed March 17, 2019).

[b0230] Statistics Canada. (2019h). Table 38-10-0020-01 – Parks and green spaces [Data file]. Retrieved from https://www150.statcan.gc.ca/t1/tbl1/en/cv.action?pid=3810002001 (accessed March 16, 2019).

[b0235] Statistics Canada. (2019i). Table 38-10-0032-01 – Disposal of waste, by source [Data file]. Retrieved from https://www150.statcan.gc.ca/t1/tbl1/en/tv.action?pid=3810003201 (accessed March 16, 2019)

[b0240] Sunstein C., Thaler R. (2008). Nudge: Improving decisions about health, wealth, and happiness.

[b0245] The 169 Commandments. (2015, March 28). The Economist.

[b0250] United Nations (2018). The Sustainable Development Goals report 2018.

[b0255] United Nations Children’s Fund (UNICEF) (2018). Progress for every child in the SDG era.

[b0260] United Nations Educational, Scientific and Cultural Organization (UNESCO) Institute for Statistics. (2017). Sustainable Development Goal 4 Indicators: Adjusted net enrolment rate, one year before the official primary entry age [Data file]. Retrieved from http://uis.unesco.org/indicator/sdg4- sdg_4-target4_2-target4_2_2 (accessed May 8, 2017).

[b0265] United Nations Population Division, Department of Economic and Social Affairs (U.N.-DESA). (2017). File POP/1-1: Total population (both sexes combined) by region, subregion and country, annually for 1950-2100 (thousands) [Data file]. World population prospects: The 2017 revision. Retrieved from https://population.un.org/wpp/ (accessed March 17, 2019).

[b0270] United Nations Statistics Division. (2019). SDG indicators global database [Data file]. Retrieved from https://unstats.un.org/sdgs/indicators/database/ (accessed March 14, 2019).

[b0275] World Bank (2018). Atlas of Sustainable Development Goals 2018: World Development Indicators.

[b0280] World Bank. (2019). World Development Indicators [Data file]. Retrieved from https://datacatalog.worldbank.org/dataset/world-development-indicators (accessed March 17, 2019).

[b0285] World Data Lab. (2019). World poverty clock [Data file]. Retrieved from http://worldpoverty.io/ (accessed March 18, 2019).

[b0290] World Health Organization (2017). World health statistics 2017: Monitoring health for the SDGs, Sustainable Development Goals.

